# Mid and Near-Infrared Reflection Spectral Database of Natural Organic Materials in the Cultural Heritage Field

**DOI:** 10.1155/2018/7823248

**Published:** 2018-10-01

**Authors:** Claudia Invernizzi, Tommaso Rovetta, Maurizio Licchelli, Marco Malagodi

**Affiliations:** ^1^Arvedi Laboratory of Non‐Invasive Diagnostics, CISRiC, University of Pavia, Via Bell'Aspa 3, 26100 Cremona, Italy; ^2^Department of Mathematical, Physical and Computer Sciences, University of Parma, Parco Area delle Scienze, 7/A, 43124 Parma, Italy; ^3^Department of Physics, University of Pavia, Via Bassi 6, 27100 Pavia, Italy; ^4^Department of Chemistry, University of Pavia, via Taramelli 12, 27100 Pavia, Italy; ^5^Department of Musicology and Cultural Heritage, University of Pavia, Corso Garibaldi 178, 26100 Cremona, Italy

## Abstract

This study presents mid and near-infrared (7500-375 cm^−1^) total reflection mode spectra of several natural organic materials used in artworks as binding media, consolidants, adhesives, or protective coatings. A novel approach to describe and interpret reflectance bands as well as calculated absorbance after Kramers-Kronig transformation (KKT) is proposed. Transflection mode spectra have represented a valuable support both to study the distorted reflectance bands and to validate the applicability and usefulness of the KK correction. The aim of this paper is to make available to scientists and conservators a comprehensive infrared reflection spectral database, together with its detailed interpretation, as a tool for the noninvasive identification of proteins, lipids, polysaccharides, and resins by means of portable noncontact FTIR spectrometers.

## 1. Introduction

For about ten years, modern heritage science community has been moving toward the development of nondestructive and noninvasive diagnostic methodologies that do not require any removal of small fragments from precious or unique works of art and preferably enable in situ investigations [1–4]. Furthermore, the omission of invasive sampling makes the measurements repeatable at any time, thus allowing the potential monitoring of materials conservation state over the years and the overcoming of the limit of poor representativity typical of microdestructive analyses [5].

Fourier transform infrared spectroscopy (FTIR) is one of the most important analytical techniques for the identification of organic and inorganic chemical compounds on the basis of the functional group characterization. Until recently, infrared spectroscopy applied to the study of Cultural Heritage artefacts has been mainly used in either transmission, attenuated total reflection (ATR) or diffuse reflection (DRIFT) modes [6–8]. These techniques are very accurate and precise, giving a truthful characterization of the single specimen, at the expense of the need for sampling or the contact with the surface. In order to gradually reduce the use of sampling methods to the benefit of the artwork integrity, the last decades have seen an increasing use of noninvasive infrared reflection techniques thanks to the development in the infrared instrumentation technology. Portable noninvasive FTIR spectrophotometers work in total reflection mode by means of fiber optics [9–11] or, just more recently, reflection mode accessories [12–14]. Yet the main drawback is related to the interpretation of the reflectance spectral data, which can present large distortions of band shape, absorption frequency, and intensity compared to transmittance spectra. These anomalies are due to several factors, such as absorption (*k*) and refraction (*n*) indices and surface roughness, which affect the extent of specular (or surface) and diffuse (or volume) reflection contributions. In particular, the specular component is ruled by Fresnel's law giving rise to first-derivative-like spectral bands from materials with* k* < 1 and/or to Reststrahlen (or inverted) bands when* k *≫ 1, while the diffuse component appears as features that are very similar to those collected in transmission mode except for some differences in terms of relative band intensities [6, 15]. It is worth reporting that the application of specific data-processing algorithms, such as the Kramers-Kronig transformation (KKT), can give accurate and reliable results on condition that specific required conditions are correctly fulfilled [16–18].

As regards natural organic materials composing the art objects, existing mid-infrared (MIR) reflection studies are limited to their interaction, as binding media, with inorganic pigments [9, 19, 20] or to very specific fields of interest [21]. A valuable reflectance spectral database in the near-infrared (NIR) region has been set up [1]. In order to correctly interpret infrared reflection spectra of natural organic components in the expanded region of MIR and a portion of NIR, we considered the building of a reflectance spectral database as mandatory. This necessity is mainly due to the fact that reflection spectra cannot be generally identified using the available archives of spectral signatures acquired in transmission or ATR modes.

The paper here presented aims to fill this gap, by providing a detailed interpretation of mid (4000-375 cm^−1^) and near (7500-4000 cm^−1^) infrared total reflection mode spectra of sixteen pure, nonaged natural organic compounds used in artworks as binding media, consolidants, adhesives or protective coatings. Firstly, a description of mid-IR absorption bands acquired in transflection mode (TR) was given. In TR spectrometry, the beam is transmitted through a surface film, whose thickness must be on the order of one-half wavelength or more, and reflected from a metallic surface. Following these conditions, the collected spectrum is quite similar to the transmission spectrum of the material and the bands are considered absorption ones. These data represented a crucial starting point for the study and the interpretation of the distorted total reflectance spectral behavior in the MIR region, as well as having made available an accurate, direct visual comparison in frequency and lineshape between absorption and reflection bands. TR spectra were then used to assess the applicability and usefulness of the Kramers-Kronig correction for an easier interpretation of reflectance bands. For the sake of completeness, the interpretation of reflection spectral signals in the restricted portion of NIR region was given, as added value for the identification of the substances. Final output of this work is to provide a comprehensive database of FTIR reflectance spectra to scientists and conservators as a tool for the noninvasive identification of natural organic materials belonging to the classes of proteins, lipids, polysaccharides, and resins. In conclusion, it is worth emphasizing that the paper represents a fundamental, preliminary study for tackling the subsequent interpretation of the more complex mixtures, layered systems or aged materials occurring in real cases.

## 2. Materials and Methods

### 2.1. Reference Materials

Sixteen commercially available materials (Kremer Pigmente GmbH & Co., Aichstetten, Germany) were selected according to their widespread use in art. The reference compounds belong to the classes of polysaccharide (cellulose 63600; Arabic gum 63300), lipid (carnauba wax 62300; beeswax 62200; shellac wax 60550), proteinaceous (hide glue 63020; bone glue 63000; casein 63200) and natural resinous (sandarac 60100; manila copal 60150; colophony 60310; Venetian turpentine 62010; dammar 60001; mastic 60050; shellac 60480; dragon's blood 37000) materials.

### 2.2. Sample Preparation

In order to prepare the samples for the infrared analyses, each substance was dissolved in a proper solvent (20% w/v): distilled water for animal glues, cellulose and Arabic gum, ammonium hydroxide for casein, and absolute ethanol for all resins with the exception of Venetian turpentine for which 1-propanol was required. The solutions were then purified using a polyethylene filter funnel (stitch size of 60 *μ*m). Waxes were simply heated in water bath until reaching the melting point (60-90°C). With the aim of collecting mainly the specular component and minimizing the diffuse one in the total reflection analysis, bulk samples with flat surface were prepared using the procedure described in a preliminary paper [23]. To compare and prove the accuracy of the method, transflection mode spectra were acquired from a thin film laid on a smooth reflective substrate (i.e., aluminum foil).

### 2.3. Portable FTIR Reflectance Spectroscopy

Both total reflection and transflection mode FTIR spectra were recorded using the Alpha portable spectrometer (Bruker Optics, Germany/USA-MA) equipped with a SiC globar source, a permanently aligned RockSolid interferometer (with gold mirrors) and a DLaTGS detector. Measurements were performed at a working distance of 15 mm by an external reflectance module with an optical layout of 23°/23°. Pseudoabsorbance spectra [log(1/R); R = reflectance] were acquired between 7500 and 375 cm^−1^ from areas of about 5 mm in diameter, at a resolution of 4 cm^−1^ and with an acquisition time of 1 min. Spectra from a gold flat mirror were used as background. An average of three spectra for each sample was carried out. Mid-infrared total reflection mode spectra were transformed to absorbance spectra by applying the Kramers-Kronig algorithm (included in the OPUS 7.2 software package). A large negative band can appear in the corrected reflection spectra around 3600 cm^−1^, as an artefact of KK transformation [13].

### 2.4. Spectral Analysis

The extent of specular reflection contribution turned out to be predominant over the mid-IR region in all acquired total reflection mode spectra resulting in a derivative-like band profile. The specular reflection bands have been discussed by the functional group giving rise to characteristic slope, maximum position and shape. These derivative-shaped bands are correlated to their corresponding KK transformed bands, which are superimposable, in turn, on the absorption signals acquired in transflection mode and whose frequency attribution is widely known by literature [24–26]. The fact that the KK corrected and TR spectra are comparable means that the required conditions for a successful Kramers-Kronig transformation have been correctly fulfilled, and confirms the specular reflection as the main collected contribute of the total reflection mode spectra in the mid-IR region. From a comparative evaluation of the collected spectra, we can see that the steeper the slope of a derivative-shaped band is the sharper the lineshape of the corresponding absorption band gets, whereas slope changes mean the presence of multiple bands. Instead, the inflection point (i.e., the point on a curve at which the concavity changes) of specular reflectance bands corresponds to the band maximum of the absorption ones. However, the difficulty often detected in determining the precise point of inflection has made it more relevant to refer to the maximum of specular reflectance bands, the wavenumber value resulting up-shifted compared to that one of absorbance bands. On the other hand, specular distortions due to the Reststrahlen effect have not appeared in the analyzed spectra, as expected by low absorption coefficient materials. As regards near-IR region, combination and overtone bands were not subjected to spectral anomalies; because of the small absorption coefficients, the diffuse contribution results, indeed the dominant factor, in determining the behavior of the NIR reflection bands [6, 27].

## 3. Results and Discussion

In this section, the detailed analysis of the spectra recorded in transflection and total reflection modes from the natural organic materials, grouped into four classes, will be proposed and discussed.

### 3.1. Proteinaceous Materials

Proteins are macromolecules made up of one or more unbranched chains of amino acids, which are joined together by peptide bonds between the carboxyl and amino groups of adjacent amino acids residues. Concerning the analyzed materials, casein is a phosphoprotein complex that is precipitated by the acidification of skimmed milk whereas animal glues are obtained by dissolving collagen, the fibrous protein of connective tissues in animals which contains three polypeptide *α*-chains in a triple helix conformation. In art and art conservation, they have been mainly used as binding media, consolidants and adhesives [28] and, less frequently, in protective treatments on different material typologies (e.g., stones, fossils) [29].

**Figure 1:**
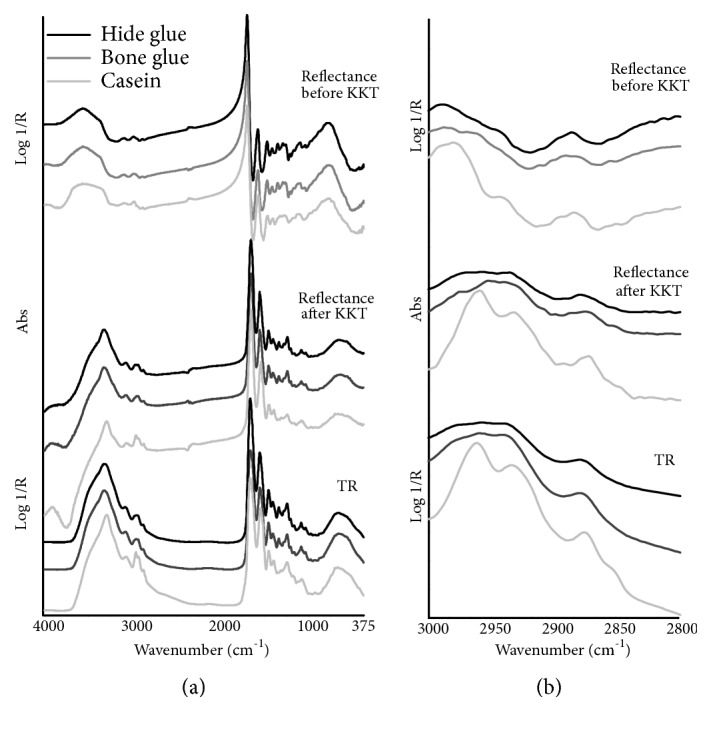
FTIR spectra in the mid-infrared region of the studied proteinaceous materials (black line: hide glue, gray line: bone glue, light gray line: casein) using different analysis modes. From top to bottom: total reflection mode before the KKT correction, total reflection mode after the KKT correction, and transflection mode. Spectral regions of (a) 4000-375 cm^−1^ and (b) CH stretching bands.

In TR mode, the mid-IR spectral range of the selected proteinaceous materials is characterized by consistent recognizable absorption peaks (Figure 1(a)). A typical stair-step pattern is formed by the amide I band in the region of 1650 cm^−1^, due to the strong carbonyl stretching vibration (C=O), then the amide II band near 1550 cm^−1^ as a combination of C-N stretching and N-H bending vibrations and finally the CH bending vibration occurring near 1450 cm^−1^, occasionally referred to as an amide III. The asymmetrical N-H stretching vibration occurring near 3300 cm^−1^ and the first overtone of amide II band positioned at 3080 cm^−1^ both appear as peaks on the broader O-H stretching band that overlaps this region. Additionally, the methyl (CH_3_) and methylene (CH_2_) groups produce asymmetric stretching vibrations respectively at 2960 and 2935 cm^−1^ and small symmetric stretching bands respectively at 2875 and 2850 cm^−1^. It should be noticed that these CH stretching bands appear better resolved in the spectrum of casein, with CH_3_ groups of greater intensity than CH_2_ groups, whereas both bone and hide glues show CH asymmetric stretches as broad overlapping bands with the CH_2_ symmetric stretching very difficult to be discerned because of its weakness (Figure 1(b)). Other small CH bending vibrations are found in the 1400-1300 cm^−1^ region as well as several C-O vibrations occur from 1250 to 1000 cm^−1^.

In total reflection mode, the mid-IR collected spectra (Figure 1(a)) display an intense and narrow maximum of the amide I band at 1698 cm^−1^, appearing predominant over the mid-infrared range, followed with decreasing intensity by the amide II and amide III bands whose sharp maxima are positioned at 1578 and 1470 cm^−1^, respectively. In the 3800-3200 cm^−1^ region, the lineshape of the *ν*_as_N-H band is characterized by a steep slope with a wide-ranging maximum near 3370 cm^−1^ while the O-H vibration produces a broad band having a slight slope and an unstructured maximum near 3570 cm^−1^. The region comprising the overtone of the amide II band and the CH stretches shows low-intensity bands with the maxima placed respectively near 3095, 2985, 2945, 2885 and 2855 cm^−1^. As observed also in TR mode, casein slightly differs from animal glues in the *ν*CH spectral range here resulting in a major slope and more structured maxima of the band lineshape (Figure 1(b)). Concerning the more pronounced intensity difference between the CH_3_ and CH_2_ bands in the spectrum of casein compared to animal glues, this could be ascribable to the presence in the former of a higher percentage amount of amino acids containing two methyl groups each (i.e., valine, leucine and isoleucine). Some other small differences can be detected in the low-wavenumber region from 1350 to 1000 cm^−1^, where less-structured and broader C-O and *δ*CH band maxima characterize casein compared to animal glues. The experimental wavenumbers corresponding to the maxima of the mid-IR total reflection mode bands of proteinaceous materials and their assignment are summarized in Table 1. After applying the KK correction, an accurate match in position and shape between corrected reflectance and transflectance bands can be observed (Figure 1).

**Figure 2:**
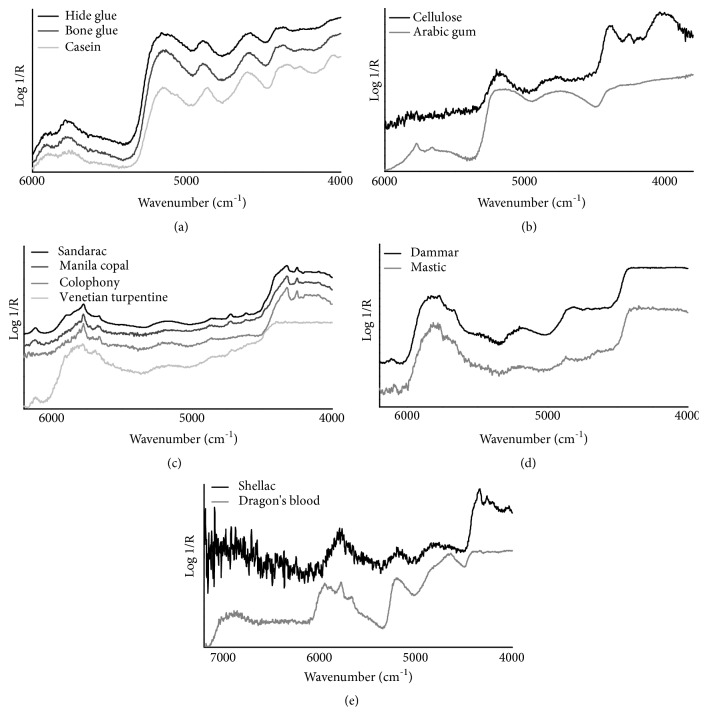
FTIR total reflection mode spectra in the near-infrared region of the studied (a) proteinaceous materials, (b) polysaccharide materials, (c) diterpenoid resins, (d) triterpenoid resins, and (e) not (exclusively) terpenoid-based resins.

Regarding the near-IR region, all three total reflection mode spectra exhibit a similar absorption pattern (Figure 2(a)). The first overtones of the asymmetric and symmetric CH_2_ stretching modes occur respectively at 5900 and 5775 cm^−1^, whereas the combination bands *ν*(OH) + *δ*(OH) and 1st overtone *ν*(C-O) amide I + amide II are visible near 5160 and 4600 cm^−1^ respectively [1]. As expected after observing the fundamental vibrations in the mid-infrared range, casein shows slightly more defined shapes of the *ν*_a_(CH_2_)+*δ*(CH_2_) and *ν*_s_(CH_2_)+*δ*(CH_2_) combination bands respectively at 4375 and 4260 cm^−1^, if compared to animal glues. Moreover, the dairy protein exhibits more resolved bands at 4865 cm^−1^, due to the *ν*(NH)+*δ*(NH) combination band, and near 4055 cm^−1^ which could be attributed to the CH functional group as a combination or overtone band [30].

### 3.2. Lipid Materials

The use of lipids in art objects is essentially limited to waxes and drying oils, respectively made up of nonglyceryl and glyceryl esters. However, only the physical properties of the waxes fulfilled the required sample preparation conditions for reflection analysis. Associated with long-chain nonglyceryl esters are one or more of the following: free fats or wax acids, alcohols, sterols, ketones and aliphatic hydrocarbons. These components vary greatly according to the origin of the wax, which can be vegetal (e.g., carnauba wax), animal (e.g., beeswax, shellac wax) or mineral (e.g., ceresin). Since ancient times, they have been employed in art conservation as adhesives and surface coatings, and particularly as waterproofing agents thanks to their hydrophobic properties [28].

**Figure 3:**
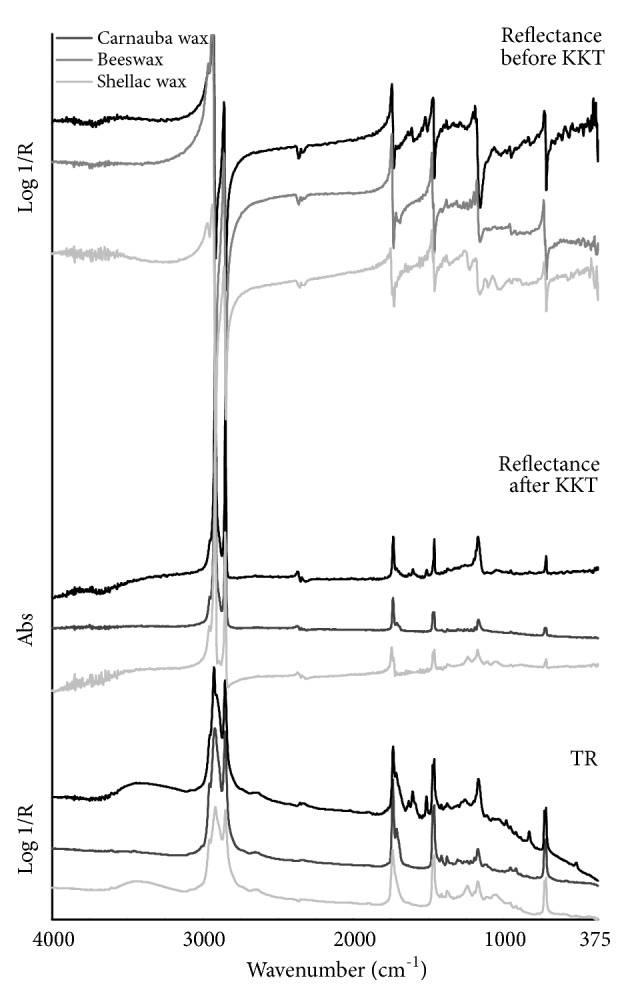
FTIR spectra in the mid-infrared region of the studied lipid materials (black line: carnauba wax, gray line: beeswax, light gray line: shellac wax) using different analysis modes. From top to bottom: total reflection mode before the KKT correction, total reflection mode after the KKT correction, and transflection mode.

In TR mode, the MIR spectra collected from the three waxes (Figure 3) display the characteristic sharp bands produced by the many CH_2_ groups: the predominant asymmetric and symmetric stretches near 2920 and 2850 cm^−1^, and the peak splitting of scissoring and rocking vibrations into doublets respectively at 1472/1462 cm^−1^ and 730/720 cm^−1^ which indicates the semicrystalline structure of the waxes [21, and references therein]. The CH_3_ methyl end groups of pure, long-chain hydrocarbons produce stretching bands near 2955 and 2905 cm^−1^. Ester and acid/ketone groups account for the C=O stretching bands respectively at 1735 and 1715 cm^−1^, appearing as an overlapping band in the shellac wax spectrum, and for the C-O bands in the 1172 cm^−1^ region. Additional characteristic sharp peaks of the carnauba wax are found at 1635, 1605, 1515, 832 and 520 cm^−1^ whereas the shellac wax spectrum shows a further well-resolved band in the region of C-O vibrations at 1240 cm^−1^ and a broad band near 1060 cm^−1^.

**Figure 4:**
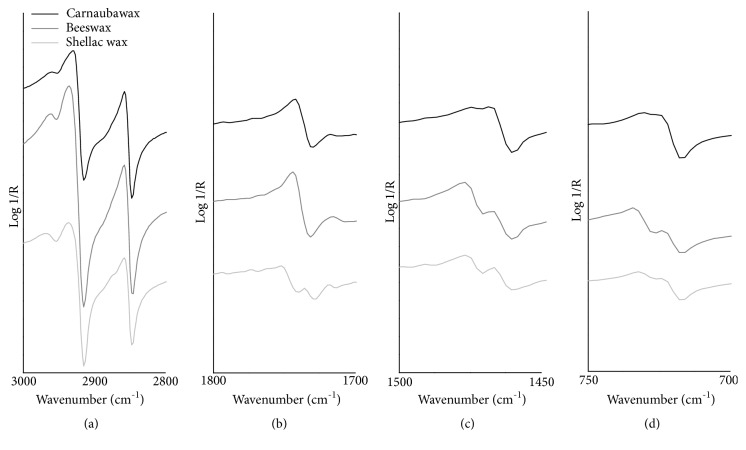
FTIR total reflection mode spectra (before the application of the KKT correction) of the studied lipid materials (black line: carnauba wax, gray line: beeswax, light gray line: shellac wax) in the regions of (a) CH stretching, (b) C=O stretching, (c) CH_2_ scissoring, and (d) CH_2_ rocking.

In total reflection mode, all acquired mid-IR spectra (Figure 3) exhibit sharp maxima of the CH_2_ stretching bands at 2935 and 2858 cm^−1^. These bands are characterized by a very steep slope of the lineshape and appear predominant over the mid-infrared range because of their intensity. The asymmetric CH_3_ stretching mode produces a wide and low-intensity band maximum near 2965 cm^−1^ while the symmetric methyl band is difficult to be discerned in these spectra. This *ν*CH region is clearly shown in Figure 4(a). Beeswax and shellac wax spectra show two close narrow maxima both of CH_2_ scissoring and rocking bands respectively at 1478/1468 and 735/725 cm^−1^, corresponding to the previously described absorption doublets (Figures 4(c) and 4(d)). Conversely, the peak splitting of these bands does not appear in the spectrum of the carnauba wax and this is confirmed observing the corresponding KK corrected spectrum. The absence of these doublets is indicative of a loss of the crystal structure of hydrocarbon chains during the bulk sample preparation procedure [31]. The C=O stretching vibrations produce a high slope ester band having the sharp maximum placed at 1745 cm^−1^ in the beeswax and carnauba wax spectra and at 1752 cm^−1^ in the spectrum of shellac wax, and a weaker acid/ketone band with the maximum respectively at 1716 and 1718 cm^−1^ (Figure 4(b)). Concerning this spectral region, shellac wax shows an additional well-defined narrow C=O band (acids/ketones) with the maximum being positioned at 1735 cm^−1^. Another band characterized by the steep slope of the lineshape, with the maximum positioned at 1183 cm^−1^, is due to C-O vibration. Those additional features detected in TR mode show the reflection maxima at 1612, 1522 and 832 cm^−1^ in carnauba wax and near 1250 and 1075 cm^−1^ in shellac wax. The experimental wavenumber values corresponding to the maxima of the mid-IR total reflection mode bands of lipid materials and their suggested assignment are summarized in Table 2.

In the NIR region, only overtones from methylenic stretching at 5770 and 5660 cm^−1^ can be clearly detected in total reflection mode spectra whereas combination bands from *ν* and *δ*CH_2_ do not occur except as broad and unresolved features. It is worth noting that NIR spectra, here not shown in figure, do have a prominent noise that could make it difficult to identify the characteristic bands.

### 3.3. Polysaccharide Materials

Polysaccharides are polymers made up of many monosaccharide units joined together by glycoside bonds, and include cellulose, starch, honey and plant gums. As regards the analyzed materials, cellulose consists of high molecular weight polymer of D-glucose with *β*(1-4)-glycosidic bonds, whereas the long-chain polymers of plant gums, exudates from several species of plants or extracted from the endosperm of some seeds, are made of aldopentoses, aldohexoses and uronic acids which condense through glycosidic bonds. Arabic gum (exuded by* Acacia senegal* and* Acacia seyal*) is not only one of the most important representatives of the plant gums' family in the field of artistic and historic works, it is also of broader economic relevance and therefore produced in rather large quantities [32, 33]. Over the time, polysaccharides have widely been used in art and art conservation as painting media and sizing agents as well as adhesives [22] and, to a lesser extent, in consolidation and protective treatments [34].

**Figure 5:**
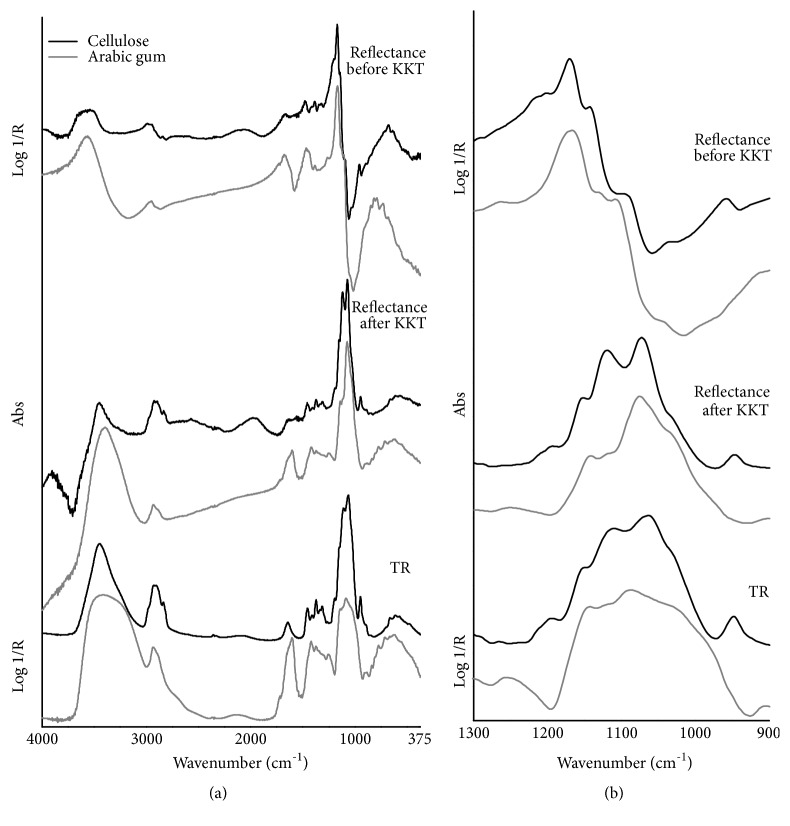
FTIR spectra in the mid-infrared region of the studied polysaccharide materials (black line: cellulose, gray line: Arabic gum) using different analysis modes. From top to bottom: total reflection mode before the KKT correction, total reflection mode after the KKT correction, and transflection mode. Spectral regions of (a) 4000-375 cm^−1^ and (b) C-O stretching bands.

The mid-infrared TR spectra of cellulose and Arabic gum (Figure 5(a)) display the characteristic IR pattern for the polysaccharides produced by the high proportion of O-H groups bound to the carbons. Indeed, both spectra show two strong, broad stretching bands: one in the 3400-3300 cm^−1^ region due to O-H groups and the other at about 1080 cm^−1^ due to C-O groups. A moderately strong band found at 1650 cm^−1^ in cellulose and at 1610 cm^−1^ in Arabic gum (with asymmetric lineshape) is partially associated with intramolecular bound water and partially due to the presence of a carboxyl group, whereas the only gum spectrum presents an additional very weak, sharp peak at 1720 cm^−1^ which is attributed to the C=O stretching of an ester-containing component. The lineshape of CH stretching and bending absorptions, respectively, found in the 3000-2800 and 1480-1300 cm^−1^ regions, appears sharper and more resolved in cellulose compared to Arabic gum, even though this is generally true for all spectral features. Moreover, a further well-defined peak at 950 cm^−1^ can be ascribed to the CH rocking vibration which occurs in cellulose.

In total reflection mode, the most significant region of the MIR spectra (Figure 5(a)) corresponds to the C-O vibrations: both the polysaccharides display indeed an intense and narrow band maximum at 1171 cm^−1^, followed with decreasing intensity by well-defined peak maxima positioned at 1144 and 1100 cm^−1^ in cellulose and at 1108 in Arabic gum. The lineshape of all these combined bands is characterized by a very steep slope, thus resulting in sharp absorptions after the application of the KK logarithm. This region is more clearly shown in Figure 5(b). As regards the lower wavenumber range, the well-resolved CH vibration band occurring in cellulose at 958 cm^−1^, already highlighted at 950 cm^−1^ in the TR spectrum, can be considered a diagnostic feature for the distinction of the analyzed polysaccharides. The O-H groups produce a moderately strong band which appears more marked in the gum than in cellulose: it is characterized by a broad band maximum near 3500 cm^−1^ and a rather slight slope of the lineshape. As already pointed out by comparison with TR spectra, the CH stretching and bending regions of cellulose turn out to have more resolved features compared to those of Arabic gum (for details, see Table 3), and this is also confirmed by their corresponding KK transformed spectra. On the contrary, the combined C=O and H-O-H (intramolecular water) vibrations give a response which is more intense in the gum than in cellulose. Concerning this region (1700-1550 cm^−1^), the reflectance spectrum of Arabic gum displays an overlapping band characterized by two different slopes which give rise to a sharp peak at 1605 cm^−1^ and a shoulder near 1645 cm^−1^ in the corresponding KK spectrum. Furthermore, it is possible to notice that only a weak and poorly resolved feature near 1730 cm^−1^ (C=O vibration) occurs in the reflectance spectrum of Arabic gum, whereas a small sharp peak has been well distinguished using the TR technique. The detailed experimental wavenumber values corresponding to the maxima of the mid-IR total reflection mode bands, and the relative suggested assignment, are reported in Table 3.

As regards NIR region (Figure 2(b)), the investigated total reflection mode spectra are characterized by different features except for the common broad bands respectively found near 5190 cm^−1^, due to the combination of O-H stretching and H-O-H bending, and near 4760 cm^−1^ which is attributed both to the overtone of C-O stretching (including C=O and C-O) and to the combination of O-H bending and C-O stretching. In addition to those ones, the spectrum of cellulose displays the O-H and C-O stretching combination band at 4400 cm^−1^, the overtone band of CH bending vibration at 4250 cm^−1^ with a possible further contribute of CH_2_ stretching and bending combination band and, finally, the combination band of CH and C-O-C stretches and C-C vibration positioned at 4010 cm^−1^. On the other hand, Arabic gum exhibits the overtones bands of CH_2_ stretching vibrations at 5775 (asymmetric) and 5660 (symmetric) cm^−1^ whereas the combination bands produced by the same CH_2_ vibrations, which are expected to be found in the 4300-4200 cm^−1^ region [30], do not clearly appear.

### 3.4. Natural Resinous Materials

Natural resins are polymers exuded from plants or secreted by insects: although their chemistry is diverse, most are mainly composed of terpenoids, which are formally considered compounds made up of units of isoprene. Since ancient times natural resins, alone, or in mixture with other substances, have been used extensively by artists and conservators as varnishes, consolidants, adhesives, hydrorepellents and sealing agents thanks to their intrinsic properties [22, 28]. According to their chemical composition, this section is structured into three subdivisions: diterpenoid, triterpenoid and not (exclusively) terpenoid-based resins.

*Diterpenoid resins *Diterpenoid resins are primarily composed of mixtures of tricyclic (abietanes and pimaranes) and dicyclic (labdanes) resin acids. The botanical origin and the chemical composition of the analyzed diterpenoid resins (i.e., colophony, Venetian turpentine, sandarac and Manila copal) are listed in Table 4 [22]. For the spectral interpretation, it is worth noting that the composition of Venetian turpentine and colophony is similar, with the former containing a remarkable amount of labdane alcohols in addition to resinous acids. As far as their composition is concerned, also sandarac and Manila copal are very similar one to the other both consisting of free diterpenoids and a highly polymerized fraction of polycommunic acid.

**Figure 6:**
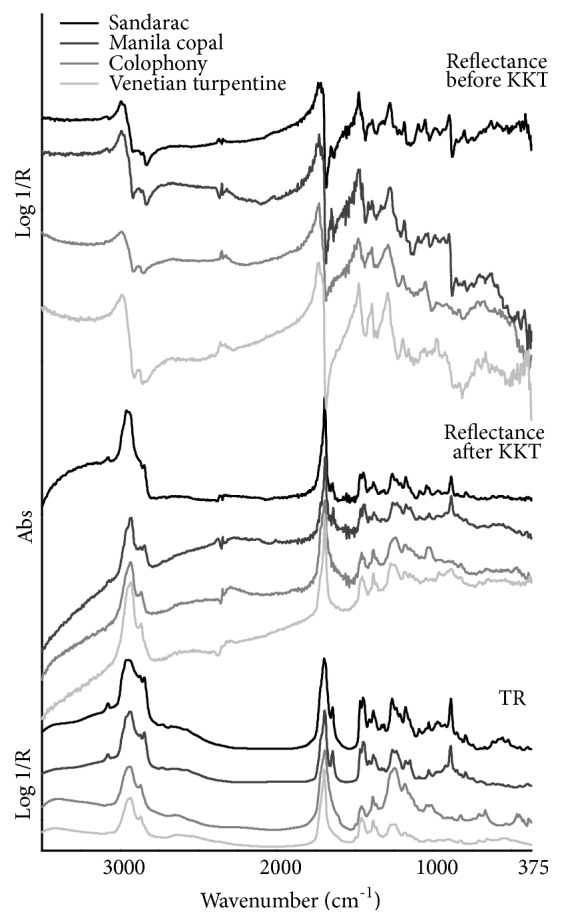
FTIR spectra in the mid-infrared region of the studied diterpenoid resins (black line: sandarac, dark gray line: Manila copal, gray line: colophony, light gray line: Venetian turpentine) using different analysis modes. From top to bottom: total reflection mode before the KKT correction, total reflection mode after the KKT correction, and transflection mode.

In TR mode, the spectra of the four diterpenoid resins show an overall similar pattern in the MIR region (Figure 6). In particular, an accurate wavenumber correspondence is observed between Venetian turpentine and colophony, on one hand, and between sandarac and Manila copal, on the other, reflecting the respective alike compositions as reported by Daher et al. [24]. All spectra are characterized by intense signals in the CH stretching region, with an overlapping band at about 2945 cm^−1^ due to the asymmetric CH_3_/CH_2_ modes and a defined peak at 2870 cm^−1^ due to the symmetric CH_3_ mode. In addition, sandarac and Manila copal exhibit a sharp band produced by symmetric CH_2_ vibrations at 2850 cm^−1^, which suggests a considerable number of CH_2_ groups occurring in these resins. Broad O-H bands are found near 3420 and 2650 cm^−1^, respectively due to the hydroxyl stretching absorption and to the O-H vibration of dimerized carboxyl groups. The C=O stretching vibration produces a characteristic sharp, strong band with the maximum falling from 1698 to 1692 cm^−1^. CH bending vibrations are found at about 1460 cm^−1^, with well-resolved features at 1468 (CH_2_ groups) and 1450 (CH_3_ groups) cm^−1^ occurring only in sandarac and Manila copal, and at 1385 cm^−1^ due to the symmetric CH_3_ mode. Moreover, several bands due both to C-OH and C-O groups of esters and acids and to CH_2_ vibrations are visible from 1300 to 900 cm^−1^, resulting in a more defined and well separated band lineshape for sandarac and Manila copal if compared to Venetian turpentine and colophony. Concerning this spectral region, colophony presents the most intense feature with respect to the other resins, falling at about 1250 cm^−1^. In addition to all these described common frequencies, it is important to notice that sandarac and Manila copal show a recognizable, characteristic system of peaks mostly related to the conjugated double bonds occurring in the side chain of communic acid, which predominantly composed the highly cross-linked fraction of these resins. These sharp, narrow and moderately strong bands are positioned at 3080 (*ν*CH from C=C bonds, appearing very weak in Venetian turpentine and colophony), 1645 (*ν*C=C) and 890 (out-of-plane bending of the exomethylene groups) cm^−1^. Furthermore, these two resins exhibit other well resolved, weak bands at 2730, 1410, 1330, 1315, 1030, 795 cm^−1^.

In total reflection mode spectra (Figure 6), the most intense band over the MIR of all these substances is produced by C=O groups of resinous acids, with the maximum falling from approximately 1730 to about 1715 cm^−1^ and the lineshape being characterized by a rapid, steep slope. As can be seen in the same figure, these groups give rise to the strongest and sharpest peak of the KK transformed spectra. Another very striking region corresponds to the *ν*CH vibrations where the asymmetric CH_3_/CH_2_ modes produce an intense band maximum near 2985 cm^−1^ followed by a steep band slope. The weak symmetric CH_3_ stretching band, with the maximum being placed near 2880 cm^−1^, appears more resolved in Venetian turpentine and colophony with respect to sandarac and Manila copal which present the additional, sharp symmetric CH_2_ stretching band maximum at 2857 cm^−1^. Moderately strong *δ*CH bands, all characterized by a rapid slope of the lineshape, present sharp maxima at around 1475 cm^−1^ and 1395 cm^−1^, in addition to which the 1455 cm^−1^ band maximum (due to CH_3_ groups) occurs in all resins with the exception of Venetian turpentine. This last further feature contributes to form the well-resolved doublet that is visible in the 1490-1420 cm^−1^ region of the KKT spectra. The C-O vibrations produce a significant medium-intensity band with the maximum falling from 1290 to 1280 cm^−1^ and gradually weaker bands with the maxima placed near 1190 and 1050 cm^−1^. On the other hand, the O-H bands cannot be easily discerned in reflection mode. As previously reported, sandarac and Manila copal exhibit some characteristic additional features related to the presence of conjugated double bonds in their composition. In detail, the weak and well-defined band with the maximum at 3085 cm^−1^, then the weak-to-moderate band with the sharp maximum at 1650 cm^−1^, and finally the moderately strong and well-resolved band having the maximum positioned at 900 cm^−1^ as well as a very steep slope of the lineshape. The experimental wavenumber values corresponding to the maxima of the mid-IR total reflection mode bands, and the relative suggested assignment, are reported in Table 5.

As regards NIR range, all reflectance spectra (Figure 2(c)) are characterized by similar pattern except for Venetian turpentine which displays no features over the 4400-4000 cm^−1^ region (a flat, steady line is here visible). In this region, indeed, the other three resins exhibit sharp, well resolved *ν*_a_(CH_2_)+*δ*(CH_2_) and *ν*_s_(CH_2_)+*δ*(CH_2_) combination bands respectively found at 4325 and 4252 cm^−1^. Common to all resins are the first overtone bands from methylenic stretching at 5775 (asymmetric) and 5665 (symmetric) cm^−1^, then the weak and broad combination band of *ν*(OH)+*δ*(OH) which is centered at around 5170 cm^−1^, and finally the poorly defined band near 4865 cm^−1^. Weak-to-moderate and well-defined bands are also found at 6120 and 4725 cm^−1^, respectively due to the first overtone of *ν*(CH_2_) cyclic and the combination of C-O and OH stretching. However, these two features do not occur in colophony. Moreover, sandarac and Manila copal display an additional weak peak at 4615 which can be attributed to the combination of *ν*(C-O)+ *ν*(CH_2_) [1].

*Triterpenoid resins* Triterpenoid resins consist of mixtures of triterpenoid molecules with mainly pentacyclic (ursanes, oleananes, lupanes and hopanes) and tetracyclic (dammaranes and lanostanes) skeletons. Table 4 reports the botanical origin and the kind of terpenoid compounds of the analyzed triterpenoid resins (i.e., mastic, dammar).

**Figure 7:**
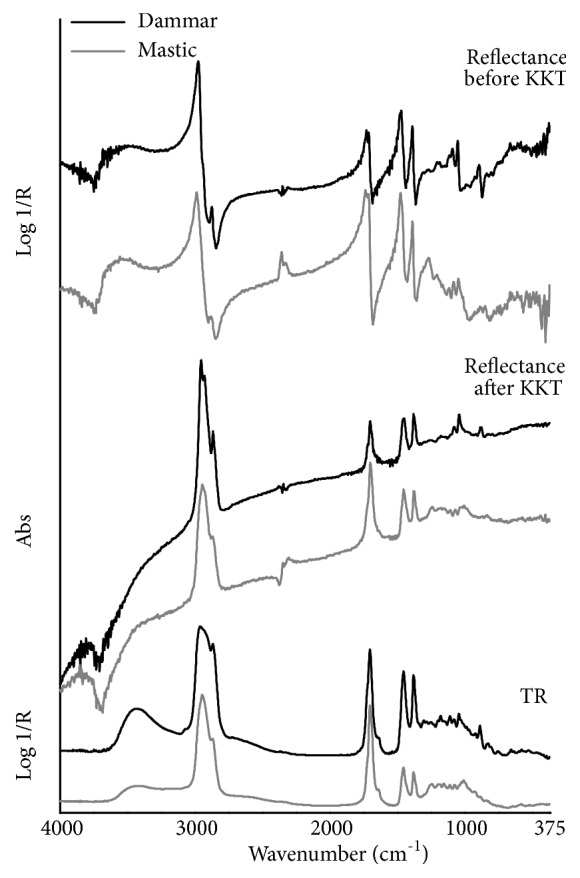
FTIR spectra in the mid-infrared region of the studied triterpenoid resins (black line: dammar, gray line: mastic) using different analysis modes. From top to bottom: total reflection mode before the KKT correction, total reflection mode after the KKT correction, and transflection mode.

The mid-IR TR spectra of the two analyzed resins are characterized by the same band frequencies, with some slight differences appearing in the low-wavenumber peaks intensity (Figure 7). Moreover, the main fundamental frequencies are in common with the diterpenoid resins, resulting in extremely similar IR profiles which make it difficult to identify the different plant resins. The acquired spectra exhibit the most intense absorptions at 2945 (a saturated, less defined signal occurs in dammar) and 2872 cm^−1^, respectively due to asymmetric CH_3_/CH_2_ and symmetric CH_3_ stretches, and at 1705 cm^−1^, due to the C=O stretching of the resinous acids. Further characteristic sharp, moderately strong peaks are produced by the CH bending vibrations at 1455 (CH_3_/CH_2_ groups) and 1380 (CH_3_ group) cm^−1^. On the other hand, the intensity of all the C-O vibrations falling in the 1300-900 cm^−1^ region appears low to very low. Another weak band, characterized by a quite broad shape, is due to the O-H stretching vibration and is centered at around 3440 cm^−1^. Moreover, the presence of carbon-carbon double bonds in the cycling ring structure or in its side chains produces the weak bands positioned at 3070 (*ν*CH from C=C bonds) and 1645 (*ν*C=C) cm^−1^, and the peak at 890 cm^−1^ (out-of-plane bending of the exomethylene groups) which appears sharp and weak-to-moderate in dammar resin. Differently from diterpenoid resins, mastic and dammar do not clearly show the O-H vibrations of dimerized carboxyl groups in the region of 2700-2500 cm^−1^.

The MIR total reflection mode spectra of mastic and dammar (Figure 7) exhibit four recognizable, strong bands each of which is characterized by very steep slope and sharp, well-resolved maximum. Their band maxima fall at about 2980 (*ν*_as_CH_3_/CH_2_), 1715 (*ν*C=O), 1475 (*δ*CH_3_/CH_2_) and 1392 (*δ*CH_3_) cm^−1^. It should be noticed that the carbonyl stretching vibration produces another well-defined, weak band maximum at about 1735 cm^−1^ that can be ascribed to oxidation products (ketones, esters, lactones) [32] whose formation could have occurred during the bulk sample preparation. This feature gives rise to a slight asymmetry toward higher wavenumbers of the C=O band in the corresponding KK transformed spectra (Figure 7), which is not observed in TR mode. Other weak and narrow band maxima are found at 2880 cm^−1^ (*ν*_s_CH_3_) and over the C-O vibrations region 1300 to 900 cm^−1^, whereas the weak-to-moderate *δ*H_2_C=C band with the lineshape having a rapid slope and the maximum being positioned at 896 cm^−1^ clearly occurs only in dammar resin. However, the other bands due to the carbon-carbon double bonds vibrations do not appear in reflection mode. The only broad band over the mid-IR range is produced by the *ν*O-H vibrations and is characterized by a slight slope of the lineshape and a maximum falling near 3500 cm^−1^; this band does not clearly occur though after the KK correction. The experimental wavenumber values corresponding to the maxima of the mid-IR total reflection mode bands, and the relative suggested assignment, are given in Table 6.

The NIR total reflection mode spectra display the first overtone bands of asymmetric and symmetric CH_3_/CH_2_ stretches, respectively near 5800 and 5760 cm^−1^, as predominant over this spectral region. Moreover, the weak first overtone band of the *ν*CH_2_ cyclic is found at 6100 cm^−1^ in dammar resin, while it is not clearly visible in mastic because of its marked spectral noise. In addition, less pronounced combination bands occur near 5180 (*ν*OH+*δ*OH) and 4850 (*ν*C-O+*ν*OH) cm^−1^. It is worth noting that the 4400-4000 cm^−1^ region, where the combination bands of CH stretching and bending vibrations typically occur [1], does not show any features (a flat, steady line is here visible, as found in Venetian turpentine). The NIR total reflection mode spectra are reported in Figure 2(d).

*Not (Exclusively) Terpenoid-Based Resins* Among those resins that are not composed only of terpenes, shellac and dragon's blood have been playing a prominent role in the field of Cultural Heritage. Shellac, derived from secretions of the lac beetle and widely used in protective coatings for wooden surfaces, paintings, metal artworks and many other objects, is a complex mixture made of mono- and polyesters of hydroxy-aliphatic and sesquiterpene acids whereas dragon's blood (botanical origin and chemical composition are reported in Table 4) has been mainly used as a coloring matter in paint, enhancing the color of precious stones and glass, marble and the wood for violins [35].

**Figure 8:**
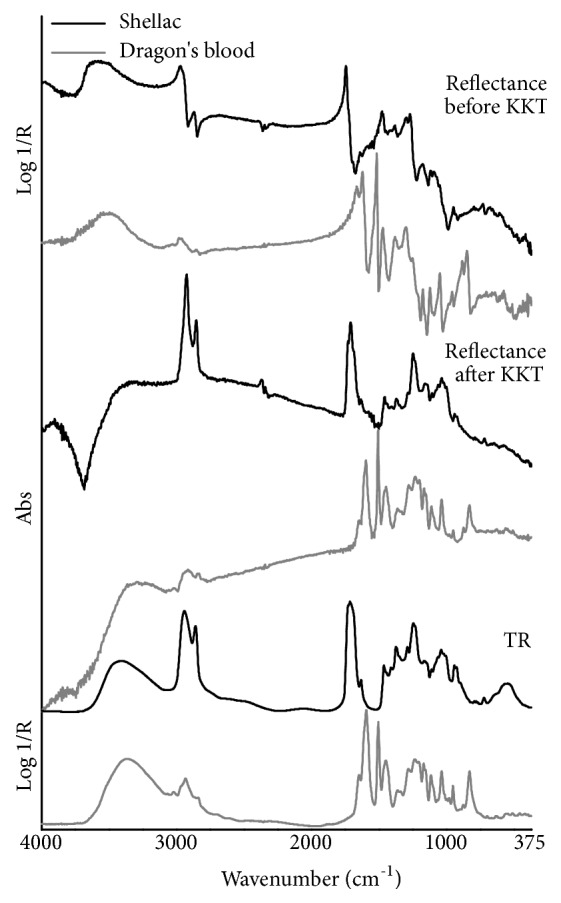
FTIR spectra in the mid-infrared region of the studied not (exclusively) terpenoid-based resins (black line: shellac, gray line: dragon's blood) using different analysis modes. From top to bottom: total reflection mode before the KKT correction, total reflection mode after the KKT correction, and transflection mode.

In TR mode (Figure 8), the MIR spectrum of shellac shows the intense, sharp CH stretching bands at 2945 (asymmetric) and 2860 (symmetric) cm^−1^ and the strong, poorly resolved doublets at 1730-1715 and 1245-1235 cm^−1^ due to the stretching of the carbonyl (from acids and esters) and the C-O groups, respectively. Medium-intensity bands are produced by CH bending vibrations lying at 1375 (CH_3_ asymmetric mode) and 1465 cm^−1^ (CH_2_ in-plane bending or scissoring), with the weak CH_3_ asymmetric mode shoulder occurring at 1448 cm^−1^, and by C-O stretching vibrations from approximately 1200 to about 1000 cm^−1^. A similar intensity characterizes the broad O-H stretching band centered at 3420 cm^−1^ and the characteristic doublet at 945-930 cm^−1^. Moreover, the carbon-carbon stretching vibrations produce a weak-to-moderate and sharp olefinic band at 1636 cm^−1^ as well as a weak and well-defined C-C band at 725 cm^−1^ from partially crystalline long-chain hydrocarbons. On the other hand, the spectrum of dragon's blood is characterized by many sharp, well-resolved bands in the fingerprint region. The strongest peaks over this region are produced by the stretching vibration of aromatic carbon-carbon double bonds at 1596 and 1507 cm^−1^, with the band at 1450 cm^−1^ appearing less pronounced [25]. Bands of medium intensity are found at 1650 cm^−1^, due to the C=C stretching mode, in the region of the C-O stretching vibrations at 1235-1210, 1172-1160 and 1115 cm^−1^, and, finally, from approximately 1000 to about 800 cm^−1^ where in-plane aromatic CH bending vibrations occur at 1040 cm^−1^ and out-of-plane aromatic and olefinic CH bending vibrations appear at 955 and 830 cm^−1^ [36]. Toward the high-wavenumber region, a moderately strong, broad O-H stretching band lies near 3370 cm^−1^ while CH stretching vibrations produce several weak bands at 3025 (aromatic and olefinic groups), 2865 (CH_3_ groups) and 2840 (CH_2_ groups) cm^−1^ and a weak-to-moderate band at 2935 cm^−1^ (CH_2_ groups) with the shoulder of CH_3_ groups positioning at 2965 cm^−1^.

**Figure 9:**
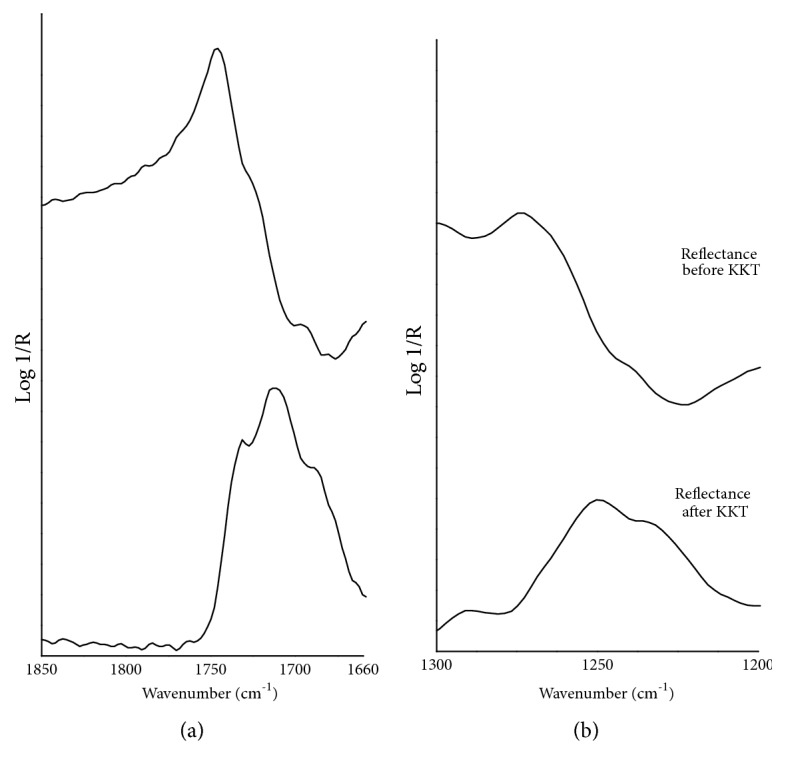
FTIR total reflection mode spectra of shellac before and after the KKT correction in the regions of (a) C=O stretching bands and (b) C-O stretching bands.

In the total reflection mode spectrum of shellac (Figure 8), the most intense features over the MIR range appear with a steep slope of the lineshape and sharp band maxima that fall at 2970 and 2873 (*ν*CH), 1747 (*ν*C=O) and 1273 (*ν*C-O) cm^−1^. It is worth noting that both C=O and C-O stretching bands present a slight slope variation along the lineshape, which is responsible for the formation of the 1731-1712 and 1250-1235 cm^−1^ doublets after the KK transformation, respectively (Figure 9). Weak-to-moderate bands are all characterized by a slighter lineshape slope with the less resolved maxima falling near 1470, 1385, 1180, 950-930 and 733 cm^−1^. Their corresponding peak assignments are reported in Table 7. The *ν*C-O bands in the 1200-1000 cm^−1^ region are partially well resolved and partially defined only by slope changes of the lineshape, as confirmed observing the KKT spectrum. The *ν*O-H band differs from all others because of its broad maximum, centered at around 3570 cm^−1^, and its slightly pronounced slope which extends from approximately 3500 to about 3200 cm^−1^. However, in correspondence of this high wavenumbers region the KK algorithm produces a flat line which is preceded by a deep falling as artefact. The total reflection mode spectrum of dragon's blood (Figure 8) presents a peculiar, distinctive fingerprint region where most of the bands have a well-resolved lineshape with steep to very steep slopes and sharp maxima (see Table 7 for the wavenumber values). As expected, the KK spectrum has the same profile of the TR one whose bands and relative assignment has been already accurately described in the paragraph above. Observing the mid-IR region over the 2000 cm^−1^, the medium-intensity O-H stretching band exhibits a wide maximum near 3500 cm^−1^ and a characteristic slightly pronounced slope, while the CH stretching vibrations produce weak-to-moderate bands with the lineshape being characterized by a few slope variations which give rise to CH_3_/CH_2_ overlapping bands after the application of the KK transformations. The detailed experimental wavenumber values of both shellac and dragon's blood, and the relative suggested assignment, are reported in Table 7.

As regards NIR region, the reflectance spectra of shellac and dragon's blood (Figure 2(e)) display a few common bands positioned at 5778 cm^−1^, due to the first overtone of asymmetric CH_2_ stretching vibration, and near 5200, 4800, and 4650 cm^−1^ which are attributed respectively to *ν*(OH)+*δ*(OH), *ν*(C-O)+*ν*(OH) and *ν*(C-O)+*ν*(CH_2_) combination bands. It should be noted that the described *ν*(C-O)+*ν*(CH_2_) band appears more intense and better resolved in dragon's blood compared to shellac, because of the likely additional contribution of the aromatic *ν*(CH)+*ν*(CC) combination band. Other common features are found in the 4500-4000 region, where the *ν*_as_(CH_2_)+*δ*(CH_2_) and *ν*_s_(CH_2_)+*δ*(CH_2_) combination bands lie respectively at 4342 and 4263 cm^−1^. These bands are strong, sharp, and well defined in the spectrum of shellac because of its characteristic aliphatic chains while they appear broad, very weak in the spectrum of dragon's blood. Moreover, in this spectral portion shellac shows another signal at 4040 cm^−1^, which is due to the third overtone of the CC bending vibration. Conversely, additional high-wavenumber bands linked to the presence of aromatic structures characterize the dragon's blood resin at about 5950 and 6900 cm^−1^, respectively attributed to the first overtone of aromatic CH stretching vibration and to the dual contribution of the first overtone of *ν*(OH) and the aromatic CH combination. Finally, it can be noticed that the first overtone band of *ν*(CH_2_) at 5668 cm^−1^ appears well defined only in the spectrum of dragon's blood, whereas the marked noise toward the high wavenumbers does not allow us to discern it in the shellac resin. It is important to point out that the interpretation of the NIR signals of dragon's blood has been based on vibration charts [30], because no band assignment has occurred in literature using the infrared spectroscopy technique.

## 4. Conclusions

The aim of the present paper is to provide a valuable analytical tool to conservators and scientists for a complete comprehension of infrared spectra acquired in total reflection mode by means of portable noncontact FTIR spectrometers. The knowledge of the reflectance spectral behavior in the mid and near-infrared region of pure standards represents, in fact, the first necessary step for subsequently tackling the more complex interpretation of aged substances, mixtures (e.g., varnishes) or layered systems occurring in real cases. Understanding when specific data-processing algorithms can be applied with accurate results represents another important issue here discussed.

In this work, we present FTIR total reflection mode spectra of sixteen pure, nonaged natural organic materials widely spread in works of art. The spectral analysis of absorption bands acquired in transflection mode has been significant, firstly, to approach the study of distorted reflection bands and, then, to evaluate the applicability of KK correction in the MIR region. Moreover, a visual comparison between reflection and absorption bands is useful to make the different spectral behavior understandable.

The study mainly focuses on the mid-IR region (4000-375 cm^−1^) where the specular reflection contribution appears predominant giving rise to a derivative-shaped spectral profile. According to low absorption coefficient materials, Reststrahlen bands do not occur. A novel, tentative approach to the discussion of specular reflectance bands compared to absorbance ones is here provided. The good wavenumber and lineshape correspondence between KK corrected and transflection bands allowed us to conclude that an accurate result from KK algorithm has been achieved for the sample references, thus confirming the specular reflection as the main collected contribute of the total reflection mode spectra in the mid-IR region. Then, the work tackles the interpretation of combination and overtone bands in the near-IR range (7500-4000 cm^−1^), where the diffuse reflection phenomenon is dominant.

Despite the possible limitation represented by artwork superficial conditions, which necessarily have to be evaluated case by case, we consider our results promising for their application in the Cultural Heritage field.

## Figures and Tables

**Table 1:** Experimental wavenumber values corresponding to the maxima of the mid-IR total reflection mode bands of proteinaceous materials and their tentative assignment.

**Experimental wavenumbers (cm** ^**-1**^ **) **	**Band assignment**
**Casein and animal glues **	
3570	*ν*OH
3370	*ν*_as_NH
3095	Overtone of combination band *δ*NH and *ν*CN (amide II)
2985	*ν*_as_CH_3_
2945	*ν*_as_CH_2_
2885	*ν*_s_CH_3_
2855	*ν*_s_CH_2_
1698	*ν*C=O (amide I)
1578	Combination band *δ*NH and *ν*CN (amide II)
1470	*δ*CH (amide III)
1400-1300	*δ*CH
1300-1000	C-O (COO^−^)

**Table 2:** Experimental wavenumber values corresponding to the maxima of the mid-IR total reflection mode bands of lipid materials and their tentative assignment.

**Experimental wavenumbers (cm** ^**-1**^ **)**			**Band assignment**
**Beeswax**	**Carnauba wax**	**Shellac wax**	
2965	2965	2965	*ν*_as_CH_3_
2935	2935	2935	*ν*_as_CH_2_
2858	2858	2858	*ν*_s_CH_2_
1745	1745	1752	*ν*C=O (esters)
		1735	*ν*C=O (acids/ketones)
1716	1716	1718	*ν*C=O (acids/ketones)
	1612		
	1522		
1478/1468	1468	1478/1468	*δ*_sciss_ CH_2_
		1250	C-O
1183	1183	1183	C-O
		1075	
	832		
735/725	725	735/725	*δ*_rock_CH_2_

**Table 3:** Experimental wavenumber values corresponding to the maxima of the mid-IR total reflection mode bands of polysaccharide materials and their tentative assignment.

**Experimental wavenumbers (cm** ^**-1**^ **)**		**Band assignment**
**Cellulose **	**Arabic gum **	
3510	3560	OH
2985		*ν*_as_CH_3_
2950	2950	*ν*_as_CH_2_
2918	2900	*ν*_s_CH_3_
2845		*ν*_s_CH_2_
	1730	*ν*C=O (esters)
1670	1665	C=O and HOH (intramolecular water)
	1620	C=O and HOH (intramolecular water)
1470	1445	*δ*CH, *ν*C-O and *δ*COH
1385	1380	*δ*CH, *ν*C-O and *δ*COH
1345		*δ*CH, *ν*C-O and *δ*COH
1320		*δ*CH, *ν*C-O and *δ*COH
	1265	*δ*CH, *ν*C-O and *δ*COH
1200		*ν*C-O
1171	1171	*ν*C-O
1144		*ν*C-O
1100	1108	*ν*C-O
958		*δ*_rock_CH

**Table 4:** Botanical origin and chemical composition of the analyzed plant resins [22].

**Order**	**Family**	**Genus (type of resin)**	**Main composition**
Coniferales	Pinaceae	*Pinus* (colophony)	Abietadienic acids, pimaradienic acids
		*Larix *(Venetian turpentine)	Abietadienic acids, pimaradienic acids, epimanool, larixol, larixyl acetate
	Araucariaceae	*Agathis* (Manila copal)	Sandaracopimaric acid, communic acid, agathic acid, abietic acid
	Cupressaceae	*Tetraclinis articulata *(sandarac)	Pimaradienic acids (sandaracopimaric acid), communic acid, totarol
Guttiferales	Dipterocarpaceae	*Hopea* (dammar)	Dammaranes (hydroxydammarenone, dammaradienol), ursanes (ursonic acid, ursonaldehyde)
Sapindales	Anacardiaceae	*Pistacia* (mastic)	Euphanes (masticadienonic acid, isomasticadienonic acid), dammaranes, oleanananes (oleanonic acid, moronic acid)
Arecales	Arecaceae	*Daemonorops *(dragon's blood)	Dracoresinotannol, dracorubin, dracorhodin, abietic acid

**Table 5:** Experimental wavenumber values corresponding to the maxima of the mid-IR total reflection mode bands of diterpenoid resins and their tentative assignment.

**Experimental wavenumbers (cm** ^**-1**^ **)**				**Band assignment**
**Sandarac**	**Manila copal**	**Colophony**	**Venetian turpentine**	
3085	3085			*ν*HC=C
2985	2985	2985	2985	*ν*_as_CH_3_/ CH_2_
2880	2880	2880	2880	*ν*_s_CH_3_
2857	2857			*ν*_s_CH_2_
1720	1730	1730	1718	*ν*C=O
1650	1650			*ν*C=C
1475	1475	1475	1475	*δ*CH_2_
1455	1455	1455		*δ*_as_CH_3_
1395	1395	1395	1395	*δ*_s_CH_3_
1280	1280	1290	1290	C-O
1190	1190	1190	1190	C-O
1050	1050	1050	1050	C-O
900	900			*δ*H_2_C=C

**Table 6:** Experimental wavenumber values corresponding to the maxima of the mid-IR total reflection mode bands of triterpenoid resins and their tentative assignment.

**Experimental wavenumbers (cm** ^**-1**^ **)**		**Band assignment**
**Mastic **	**Dammar**	
3500	3500	*ν*OH
2988	2978	*ν*_as_CH_3_/CH_2_
2880	2880	*ν*_s_CH_3_
1737	1731	*ν*C=O (ketones, esters, lactones)
1715	1715	*ν*C=O (acids)
1475	1475	*δ*CH_3_/CH_2_
1392	1392	*δ*CH_3_
1270	1270	C-O
1207	1207	C-O
1116	1116	C-O
1085	1094	C-O
1050	1055	C-O
	896	*δ*H_2_C=C

**Table 7:** Experimental wavenumber values corresponding to the maxima of the mid-IR total reflection mode bands of not (exclusively) terpenoid-based resins and their tentative assignment.

**Experimental ** **wavenumbers (cm** ^**-1**^ **)**	**Band assignment**
**Shellac **	
3570	*ν*OH
2970	*ν*_as_CH
2873	*ν*_s_CH
1747	*ν*C=O
1470	In-plane *δ*CH_2_
1385	*δ*_as_CH_3_
1273	*ν*C-O
1180	*ν*C-O
1120	*ν*C-O
1095	*ν*C-O
1075	*ν*C-O
1055	*ν*C-O
1015	*ν*C-O
1030	*ν*C-O
950-930	
733	*ν*C-C

**Dragon's blood**	
3500	*ν*OH
3030	*ν*CH (aromatic, olefinic)
2963	*ν*_as_CH
2855	*ν*_s_CH
1665	*ν*C=C, *ν*C=O
1623	*ν*C=C (aromatic)
1520	*ν*C=C (aromatic)
1473	*ν*C=C (aromatic)
1300	
1255	*ν*C-O
1218	*ν*C-O
1178	*ν*C-O
1164	*ν*C-O
1123	*ν*C-O
1052	In-plane *δ*CH (aromatic)
960	Out-of-plane *δ*CH (aromatic, olefinic)
885	Out-of-plane *δ*CH (aromatic, olefinic)
850	Out-of-plane *δ*CH (aromatic, olefinic)

## Data Availability

The data used to support the findings of this study are included within the article.
